# Biomechanical effects of cortical bone trajectory and hybrid CBT–TT fixation on fusion-site mechanics and adjacent segment loading: a finite element study

**DOI:** 10.3389/fbioe.2026.1747298

**Published:** 2026-07-20

**Authors:** Panpan Hu, Juncai Lei, Fengliang Wu, Zhongjun Liu, Feng Wei, Xiaoguang Liu

**Affiliations:** 1 Department of Orthopaedics, Peking University Third Hospital, Beijing, China; 2 Beijing Key Laboratory of Spinal Diseases, Peking University, Beijing, China; 3 Engineering Research Center of Bone and Joint Precision Medicine, Ministry of Education, Beijing, China

**Keywords:** adjacent segment degeneration, cortical bone trajectory, finite element, fusion, traditional trajectory

## Abstract

**Background:**

Posterior lumbar interbody fusion (PLIF) with pedicle screw fixation is a standard treatment for degenerative spinal disorders. However, rigid fixation may accelerate adjacent segment degeneration (ASD). Cortical bone trajectory (CBT) fixation has been proposed as an alternative to traditional trajectory (TT) fixation in lumbar fusion surgery. However, the biomechanical characteristics of CBT, particularly in comparison with hybrid CBT-TT fixation, remain incompletely understood.

**Methods:**

A validated L4-L5 finite element model was developed to compare CBT, TT, and hybrid CBT-TT fixation constructs. Two simulation phases were performed. The immediate postoperative model was used to evaluate fusion-site micromotion and strain distribution, whereas the post-fusion model was used to assess adjacent-segment range of motion (ROM), intradiscal pressure (IDP), and facet joint force.

**Results:**

All fixation constructs provided sufficient segmental stability. Compared with TT fixation, CBT fixation produced greater but controlled fusion-site micromotion and a broader strain-stimulated region. The hybrid CBT-TT construct demonstrated intermediate biomechanical characteristics between CBT and TT fixation. Following simulated solid fusion, CBT and hybrid CBT-TT fixation reduced superior adjacent-segment ROM, IDP, and facet joint forces relative to TT fixation, indicating lower adjacent-segment biomechanical loading.

**Conclusion:**

CBT fixation modified fusion-site mechanical conditions while maintaining overall construct stability. Both CBT and hybrid CBT-TT fixation reduced adjacent-segment biomechanical loading compared with TT fixation. These findings represent comparative biomechanical observations under simulated conditions and should not be interpreted as direct evidence of enhanced osseous fusion or prevention of ASD.

## 1 Introduction

Posterior lumbar interbody fusion (PLIF) combined with pedicle screw fixation remains a well-recognized surgical method for degenerative spinal disorders. However, bony fusion rates exhibit significant variability influenced by the choice of intervertebral cage material, ranging from 32% to 100% for polyetheretherketone (PEEK) cages and 53%–100% for titanium cages (Massaad et al., 2020). In addition, rigid fixation systems may contribute to adjacent segment degeneration (ASD), occasionally necessitating revision surgery. A retrospective analysis of 1,000 PLIF cases reported an ASD incidence of 9.0%, with symptom onset occurring at an average of 4.7 years postoperatively (Okuda et al., 2018b). Longitudinal data similarly demonstrated escalating risks, with symptomatic ASD rates increasing from 6% at 2 years to 31% at 10 years and reoperation rates rising from 5% to 15% over the same period in patients with L4 degenerative spondylolisthesis (Okuda et al., 2018a).

**FIGURE 1:**
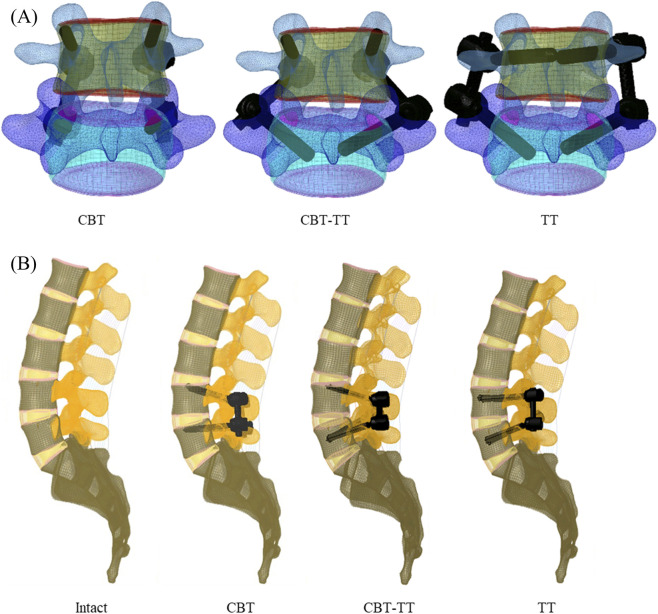
Anterior and Sagittal views of the lumbar spine models across different groups **(A)** Anterior view of the L4-L5 segment following instrumentation. **(B)**Sagittal view of the L4-L5 segment showing screw trajectories. Abbreviations: CBT, cortical bone trajectory; TT, traditional trajectory; CBT-TT, hybrid CBT and TT fixation.

To mitigate these challenges, cortical bone trajectory (CBT) screw fixation was introduced in 2009 as an alternative to traditional trajectory (TT) pedicle screws (Santoni et al., 2009). CBT screws follow a mediolateral and caudocranial path through the isthmus, pedicle, and anterolateral cortical bone, anchoring at a more medial entry point (Figure 1). This trajectory minimizes paravertebral muscle dissection, reduces intraoperative blood loss, and enhances cortical bone purchase, and provides comparable clinical efficacy to TT fixation (Matsukawa and Yato, 2017; Cofano et al., 2020; Song et al., 2014; Takenaka et al., 2017).

Despite these advantages, biomechanical studies indicated CBT fixation exhibited reduced intersegmental stiffness under axial rotation and lateral bending compared to TT screws (Matsukawa et al., 2015; Perez-Orribo et al., 2013), raising concerns about potential effects on fusion rates and ASD development. A comparative clinical study reported an 88.4% fusion rate for CBT-PLIF versus 96.3% for TT-PLIF (*P* > 0.05), a difference potentially related to CBT-associated micromotion (Sakaura et al., 2016). Paradoxically, CBT fixation demonstrated superior ASD prevention, with an incidence of only 3.2% compared to 11.0% for TT fixation (*P* < 0.05) (Sakaura et al., 2016), underscoring the need to clarify the biomechanical mechanisms underlying these findings.

Current evidence lacks a comprehensive biomechanical framework explaining how CBT’s distinct micromotion characteristics affect both fusion and ASD. To address this gap, we employed finite element (FE) analysis to quantify immediate postoperative micromotion and strain distribution among CBT, TT, and hybrid CBT-TT constructs, and to evaluate post-fusion biomechanical changes in adjacent segments.

## 2 Materials and methods

### 2.1 FE model construction

A lumbosacral spine model (L1-S1) was reconstructed from thin-slice computed tomography (CT) scans (0.625 mm thickness) obtained from a healthy 40-year-old male volunteer (height:1.75 m, weight:72 kg) without spinal abnormalities. This study was conducted using imaging data from a single healthy volunteer. The Institutional Ethics Committee of Peking University Third Hospital confirmed that formal ethical approval was not required. This study was conducted in accordance with the Declaration of Helsinki (World Medical Association, 2013). As this study involved only the analysis of imaging data, it presented no risk to the volunteer and ensured the protection of their personal information. Informed consent was obtained from the volunteer. The FE modeling process consisted of the following steps.

First, CT data were imported into Mimics v19.0 (Materialise, Belgium) for segmentation of osseous structures, generating an initial three-dimensional (3D) model in STL format. The model subsequently underwent surface refinement and topological correction in Geomagic Studio 2013 (3D Systems, United States). Intervertebral discs, endplates (1 mm), and facet cartilage (0.5 mm) were modeled in SolidWorks 2017 (Dassault Systèmes, United States) according to established anatomic parameters (Mizrahi et al., 1993). Cortical bone thickness was uniformly defined as 1 mm, while cancellous bone and posterior elements were generated through an inward offset procedure. The nucleus pulposus constituted approximately 50% of the total disc area. Material properties assigned to each structure was summarized in Table 1 and 2.

**TABLE 1:** Material properties of osseous structures.

Material	Young’s modulus (Mpa)	Poisson’s ratio	Yield stress (Mpa)	Hardening modulus (Mpa)	Hardening exponent	Failure plastic strain	Maximum stress (Mpa)	Strain rate coefficient	Density (g/cm3)
Cortical bone	14000	0.3	110	100	0.1	9.68E-03	155	1	1.1
Cancellous bone	291	0.25	1.92	20	1	1.45E-02	2.23	1	1.7
Endplate	1000	0.3	6	100	1	0.02	7.5	3	1.2
Posterior elements	3500	0.25	/	/	/	/	/	/	1.4

**TABLE 2:** Stiffness and range of motion values of the three fixation techniques.

Items	Yamamoto et al. (1989)	Intact	CBT	CBT-TT	TT
Stiffness (Nm/°)	0.440	0.457	0.500	0.520	0.561
ROM (°)	—	16.41	15.00	14.41	13.38

Abbreviations: ROM, range of motion; CBT, cortical bone trajectory; CBT-TT, hybrid CBT, and TT, fixation; TT, traditional trajectory.

### 2.2 Surgical simulation

Degenerative lumbar diseases occurred predominantly at the L4–L5 and L5–S1 levels. Given the controversies surrounding S1 CBT screw placement (Matsukawa et al., 2014), the L4-L5 segment was selected as the operative level for simulation. Bilateral partial facetectomy, annulus fibrosus resection, and nucleotomy were performed through a posteromedial approach. A 12 × 24 mm PEEK cage filled with allogeneic bone was positioned within the right central disc space, and a 5.0-mm-diameter rod was modeled. Three fixation configurations were simulated: (1) CBT screws (5 × 40 mm) inserted at L4 and L5 using a 30° cranial and 20° lateral orientation; (2) TT screws (6 × 50 mm) placed along standard pedicle trajectories; and (3) a hybrid CBT-TT construct (CBT at L4, TT at L5). The intact spine served as the non-instrumented control. Identical cage positioning, extent of facet resection, and annulus removal were maintained across all instrumented groups to isolate fixation-related effects (Figure 1).

### 2.3 Biomechanical loading protocol

The inferior surface of S1 was fully constrained in all translational and rotational degrees of freedom. To validate the intact model, a compressive preload of 500 N combined with a pure moment of 7.5 Nm was applied to the superior surface of L1 (Rohlmann et al., 2007). The predicted range of motion (ROM) was compared with previously published cadaveric data.

For comparisons among fixation constructs, a displacement-controlled loading protocol was subsequently adopted. The physiological motion envelope obtained from the intact model under the 7.5 Nm pure moment was used as the target angular displacement. Identical angular displacements were applied to the CBT, TT, and hybrid CBT–TT models during flexion, extension, lateral bending, and axial rotation, thereby eliminating differences in overall construct stiffness and allowing direct comparison of construct-specific biomechanical responses.

Immediate postoperative and post-fusion conditions were simulated separately. In the immediate postoperative model, frictional contact (coefficient of friction = 0.3) was assigned to the cage–endplate interface to permit relative micromotion (Adam et al., 2003; Yu et al., 2022). In the post-fusion model, tie constraints were assigned between the cage, bone graft, and adjacent endplates to represent an idealized solid fusion state. These two interface conditions were used to represent the early postoperative and complete fusion stages rather than the continuous biological healing process.

Micromotion was defined as the relative displacement between the superior and inferior cage–endplate interfaces within the fusion region. Adjacent-segment biomechanical responses, including ROM, intradiscal pressure (IDP), and facet joint force, were evaluated after simulated solid fusion.

### 2.4 Outcome metrics and validation

Biomechanical outcomes were assessed during two distinct simulation phases. For the immediate postoperative phase, fusion-zone micromotion and principal strain distribution were evaluated. Micromotion was quantified as the relative displacement at the cage–endplate interface. Principal strain distributions were assessed to characterize the local mechanical environment within the fusion region. For the post-fusion phase, adjacent-segment ROM, peak IDP, and facet joint force were evaluated at both the superior (L3–L4) and inferior (L5–S1) adjacent levels. Model validation was performed by comparing the ROM of the intact model under a 7.5 Nm pure moment with published cadaveric data reported by (Yamamoto et al., 1989). Agreement between predicted and experimental ROM values was considered indicative of acceptable biomechanical fidelity. A mesh convergence analysis was additionally performed using progressively refined meshes. Convergence was defined as less than 5% variation in the principal outcome measures between two successive mesh refinements. The final mesh density was selected after demonstrating numerical stability of the primary biomechanical variables.

## 3 Results

### 3.1 Model validation and biomechanical fidelity

The intact FE model demonstrated rotational stiffness of 0.457 Nm/° under 7.5 Nm moment (Table 1), closely matching the cadaveric measurements reported by Yamamoto et al. (0.44 Nm/°) (Yamamoto et al., 1989).

### 3.2 Fixation system stiffness

**FIGURE 2:**
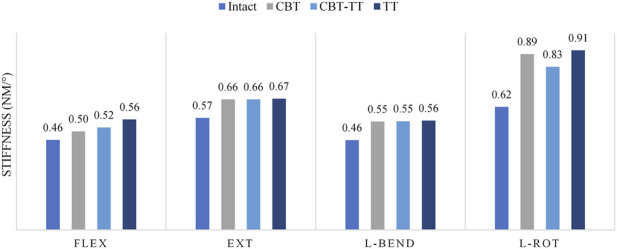
Overall construct stiffness under different loading conditions Abbreviations: CBT, cortical bone trajectory; TT, traditional trajectory; CBT-TT, hybrid CBT and TT fixation; FLEX, flexion; EXT, extension; L-BEND, left bending; L-ROT, left rotation.

All instrumented constructs (CBT, TT, and CBT-TT) exhibited significantly greater overall stiffness compared with the intact group (Table 1). The CBT (0.500 Nm/°) and TT (0.561 Nm/°) groups showed comparable global stiffness, whereas the hybrid CBT-TT construct demonstrated an intermediate stiffness value (0.520 Nm/°) (Figure 2).

### 3.3 Intersegmental micromotion and strain distribution postoperatively

**FIGURE 3:**
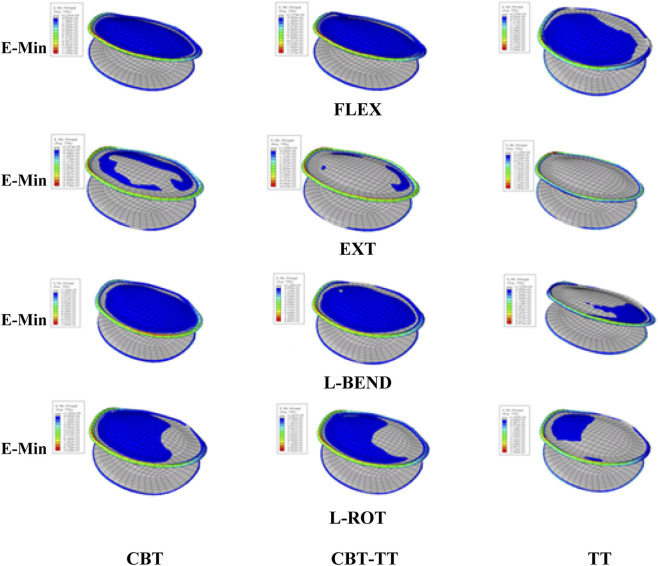
Comparison of strain-stimulated ossification zones across constructs under various motion conditions. Abbreviations: CBT, cortical bone trajectory; TT, traditional trajectory; CBT-TT, hybrid CBT and TT fixation; FLEX, flexion; EXT, extension; L-BEND, left bending; L-ROT, left rotation.

Displacement-controlled loading was applied to the superior surface of L1 to simulate flexion, extension, left bending, and left rotation up to 16°. CBT fixation permitted significantly greater micromotion than TT screws. During flexion, the CBT construct produced 77.5 μm of displacement compared with 19.7 μm for TT—a 3.9-fold increase. A similar pattern was observed during extension (119.07 μm vs. 35.5 μm), representing a 3.4-fold difference (Table 3). Strain mapping further revealed that the CBT and CBT-TT groups generated larger strain-stimulated regions across the fusion site than TT fixation (Figure 3), indicating a more favorable mechanobiological environment for promoting bone formation.

**TABLE 3:** Micromotion of the surgical segment (L4/L5) under 16° dynamic loading.

Motion (16°)	Intact	CBT	CBT-TT	TT
Flexion (μm)	349.83	77.50	45.50	19.70
Extension (μm)	649.66	119.07	106.26	35.50
Left bending (μm)	607.38	120.02	119.71	111.82
Left rotation (μm)	466.80	94.80	97.86	93.09

Abbreviations: CBT, cortical bone trajectory; CBT-TT, hybrid CBT, and TT, fixation; TT, traditional trajectory.

### 3.4 Adjacent segment biomechanical response after bony fusion

**FIGURE 4:**
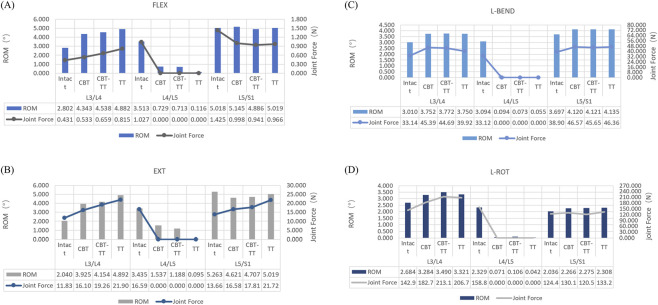
Range of motion and facet joint forces of adjacent segments under dynamic loading **(A)** FLEX; **(B)** EXT; **(C)** L-BEND; **(D)** L-ROT. Abbreviations: ROM, range of motion; FLEX, flexion; EXT, extension; L-BEND, left bending; L-ROT, left rotation.

Following simulated fusion, the CBT construct reduced upper adjacent segment (L3–L4) motion more effectively than TT. Flexion ROM decreased by 11.0% (4.343° vs. 4.882°), and extension ROM decreased by 19.8% (3.925° vs. 4.892°) (Table 4). No meaningful differences in ROM were detected in the lower adjacent segment (L5–S1) across the three fixation groups. Both the CBT and hybrid CBT-TT constructs significantly lowered peak intradiscal pressure (IDP) at L3–L4 compared with TT fixation. During flexion, IDP decreased by 9.8% in CBT and 13.9% in CBT-TT; during extension, reductions reached 18.9% and 14.4%, respectively (Table 5). Facet joint forces in the upper adjacent segment followed the order TT > CBT-TT > CBT during flexion and extension. In contrast, no substantial differences were observed in facet forces at the lower adjacent segment (L5–S1) among groups (Figure 4).

**TABLE 4:** Segmental range of motion (°) under 16° dynamic loading.

Motion (16°)	Segment	Intact	CBT	CBT-TT	TT
Flexion	L3/L4	2.802	4.343	4.538	4.882
	L4/L5	3.513	0.729	0.713	0.116
	L5/S1	5.018	5.145	4.886	5.019
Extension	L3/L4	2.040	3.925	4.154	4.892
	L4/L5	3.435	1.537	1.188	0.095
	L5/S1	5.263	4.261	4.707	5.019
Left bending	L3/L4	3.010	3.752	3.772	3.750
	L4/L5	3.094	0.094	0.073	0.055
	L5/S1	3.697	4.12	4.121	4.135
Left rotation	L3/L4	2.684	3.284	3.49	2.966
	L4/L5	2.329	0.071	0.106	0.042
	L5/S1	2.036	2.266	2.275	2.308

Abbreviations: CBT, cortical bone trajectory; CBT-TT, hybrid CBT, and TT, fixation; TT, traditional trajectory.

**TABLE 5:** Peak intervertebral disc pressure (MPa) under 16° dynamic loading.

Motion (16°)	Segment	Intact	CBT	CBT-TT	TT
Flexion	L3/L4	0.815	1.156	1.103	1.281
	L4/L5	1.679	0.062	0.048	13.240
	L5/S1	1.003	1.144	1.153	1.156
Extension	L3/L4	1.002	1.179	1.244	1.453
	L4/L5	1.309	0.068	0.053	14.610
	L5/S1	1.628	1.254	1.268	1.353

Abbreviations: CBT, cortical bone trajectory; CBT-TT, hybrid CBT, and TT, fixation; TT, traditional trajectory.

## 4 Discussion

This study provides biomechanical evidence that CBT fixation modifies mechanical parameters associated with the fusion environment and adjacent-segment loading. The main findings were as follows: CBT generated greater but controlled fusion-zone micromotion and broader strain-activated regions while maintaining overall construct stability; CBT and hybrid CBT–TT constructs reduced adjacent-segment biomechanical loading, including ROM, IDP, and facet joint forces, particularly at the superior adjacent level after simulated fusion; and the hybrid CBT–TT construct showed intermediate biomechanical behavior between CBT and TT fixation.

### 4.1 Fusion-zone micromotion and fusion-related biomechanical environment

The biomechanical microenvironment played a pivotal role in bone healing by modulating cellular behavior, differentiation, and tissue remodeling (Vordemvenne et al., 2020; Augat et al., 2021; Liu et al., 2024; Prendergast et al., 1997). The micromotion range observed in the CBT construct (≤120 μm) aligned with mechano-regulatory theory, which indicated that moderate interfacial strain facilitated angiogenesis and osteoblast differentiation (Yamamoto et al., 1989; Prendergast et al., 1997). Consistent with this, the CBT and CBT–TT constructs exhibited broader regions of osteogenic strain compared with TT fixation, supporting clinical studies reporting comparable fusion rates despite lower construct rigidity (Sakaura et al., 2016). This paradox could be explained by CBT’s unique biomechanical profile: it provided adequate global stiffness to avoid instability (0.500 Nm/°), while permitting controlled micromotion at the fusion site that stimulates callus formation. The hybrid CBT–TT construct achieved an intermediate stiffness level (0.520 Nm/°) while preserving strain-beneficial characteristics, suggesting its applicability in osteoporotic bone, revision cases, or multilevel constructs where screw purchase varies across levels.

### 4.2 Reduction of adjacent-segment biomechanical loading

ASD remains a significant challenges following lumbar fusion surgery, with rates ranging from 2% annually to as high as 30% within a decade (Jokeit et al., 2025; Matsukawa et al., 2016). Facet joint violation was one of the strongest surgical predictors of ASD (Chen et al., 2008; Matsukawa et al., 2016). Compared with TT screws, CBT’s medial entry point and shorter lever arm reduced the likelihood of cephalad facet joint violation (11.8% vs. 25%–100%) and attenuated pathological loading at the adjacent level.

In the present study, CBT fixation reduced superior adjacent-segment ROM, IDP, and facet joint force compared with TT fixation after simulated solid fusion. Similar trends were observed in the hybrid CBT-TT construct. These findings suggest that CBT-based constructs may reduce mechanical loading transferred to the adjacent segment. According to literature, the pressures are about 0.10 MPa in the lying state and about 0.3 Mpa in the sitting position (Deursen et al., 2005), so the biomechanical changes comparable to transitioning from sitting to supine positions. Such reductions correlated with the lower ASD incidence observed clinically in CBT-PLIF (3.2% vs. 11.0% in TT-TLIF) (Sakaura et al., 2016), likely mediated by two mechanisms: 1) facet joint preservation: CBT’s trajectory minimized cephalad facet violation (11.8% vs. 25%–100% in TT-TLIF) (Chen et al., 2008; Matsukawa et al., 2016), reducing iatrogenic instability; 2) load redistribution: decreased upper adjacent segment IDP (Table 4) and facet forces (Figure 4) attenuated degenerative cascades, particularly in pre-existing disc degeneration (Lau et al., 2021). Specifically, the CBT fixation groups had generated the lowest facet joint forces among the three techniques, suggesting a protective effect against facet-mediated degenerative cascades.

### 4.3 Clinical implications of the hybrid CBT-TT fixation

The hybrid CBT–TT trajectory, first described by Mobbs in 2013, was developed to combine the advantages of both constructs (Mobbs, 2013). The advantages of CBT, including less exposure during its proximal application and the retainment and fixation of the proximal joint capsule and ligaments, were thought to reduce the violation of the adjacent segments and the occurrence of ASD. Although clinical data remain limited (Mullin et al., 2016), our biomechanical results provided insights into its mechanistic rationale. The hybrid construct preserved CBT’s strain-stimulating effects while approaching TT-level stiffness, and it reduced adjacent segment IDP to a degree similar to CBT alone. These characteristics might allow surgeons to combine minimally invasive screw placement proximally with robust caudal fixation, particularly when encountering anatomical constraints such as narrow pedicles. However, due to the screw-rod connection angle required for the dual trajectory, the hybrid CBT–TT technique is most suitable for single-level fixation and may not be appropriate for multi-level constructs without specialized instrumentation.

This study systematically compared the biomechanical effects of CBT, TT, and hybrid CBT–TT fixation strategies. CBT produced greater micromotion at the index level, generating a mechanical environment conducive to fusion while simultaneously reducing adjacent segment loading. The hybrid CBT–TT technique demonstrated intermediate biomechanical behavior and may serve as a viable alternative when anatomical or surgical constraints limit the use of a uniform trajectory. Collectively, these results provide a biomechanical foundation for selecting fixation trajectories that optimize both fusion outcomes and adjacent segment preservation.

### 4.4 Limitations

Several limitations should be acknowledged. First, the model was reconstructed from CT data obtained from a single healthy 40-year-old male without degenerative or osteoporotic changes. Therefore, the findings may not be directly generalizable to elderly patients or to populations with disc degeneration, facet degeneration, endplate sclerosis, or reduced bone mineral density. Second, several modeling simplifications were adopted, including uniform cortical shell thickness, simplified ligament representations, and homogeneous isotropic material properties. Although these assumptions are commonly used in lumbar finite element studies, they may influence predicted screw stability, segmental motion, and interface stresses. Third, the cage–endplate interface was represented using a fixed friction coefficient, whereas complete fusion was simulated using tie constraints. These assumptions represent simplified mechanical endpoints and do not reproduce the continuous biological healing process or progressive changes in interface stiffness. Fourth, the model did not incorporate subject-specific paraspinal muscle forces, follower loads, time-dependent bone remodeling, cage subsidence, or long-term degeneration processes. Consequently, the present results should be interpreted as comparative biomechanical findings under standardized loading conditions rather than direct predictions of clinical outcomes. Finally, the finite element model was deterministic in nature. Parameter variability, patient-specific anatomy, and biological heterogeneity were not considered. Future investigations incorporating sensitivity analyses, probabilistic modeling, and patient-specific simulations may further improve the clinical relevance of these findings.

## 5 Conclusion

The CBT and hybrid CBT–TT fixation constructs demonstrated overall biomechanical stability comparable to TT fixation. CBT fixation produced greater but controlled fusion-zone micromotion and broader strain-activated regions at the fusion site, suggesting modification of biomechanical factors potentially relevant to the fusion environment. After simulated interbody fusion, CBT and CBT–TT constructs reduced superior adjacent-segment ROM, intradiscal pressure, and facet joint forces during flexion and extension compared with TT fixation, indicating a reduction in biomechanical loading parameters associated with ASD risk. However, because the present model did not include mechanobiological remodeling, time-dependent fusion progression, cage subsidence simulation, or long-term degeneration modeling, these findings should be interpreted as biomechanical implications rather than direct evidence of enhanced osseous fusion or prevention of adjacent segment degeneration. The hybrid CBT–TT construct may be considered in selected cases according to patient anatomy, bone quality, and surgical requirements.

## Data Availability

The original contributions presented in the study are included in the article/supplementary material, further inquiries can be directed to the corresponding author.
